# Augmented-reality swim goggles accurately and reliably measure swim performance metrics in recreational swimmers

**DOI:** 10.3389/fspor.2023.1188102

**Published:** 2023-06-07

**Authors:** Dan Eisenhardt, Aidan Kits, Pascal Madeleine, Afshin Samani, David C. Clarke, Mathias Kristiansen

**Affiliations:** ^1^ Sport Sciences—Performance and Technology, Department of Health Science and Technology, Aalborg University, Gistrup, Denmark; ^2^ Department of Biomedical Physiology and Kinesiology and Sports Analytics Group, Simon Fraser University, Burnaby, BC, Canada

**Keywords:** real-time visual feedback, swimming, performance metrics, accuracy and reliability, tracking

## Abstract

**Background:**

Swimmers commonly access performance metrics such as lap splits, distance, and pacing information between work bouts while they rest. Recently, a new category of tracking devices for swimming was introduced with the FORM Smart Swim Goggles (FORM Goggles). The goggles have a built-in see-through display and are capable of tracking and displaying distance, time splits, stroke, and pace metrics in real time using machine learning and augmented reality through a heads-up display. The purpose of this study was to assess the validity and reliability of the FORM Goggles compared with video analysis for stroke type, pool length count, pool length time, stroke rate, and stroke count in recreational swimmers and triathletes.

**Method:**

A total of 36 participants performed mixed swimming intervals in a 25-m pool across two identical 900-m swim sessions performed at comparable intensities with 1 week interval. The participants wore FORM Goggles during their swims, which detected the following five swim metrics: stroke type, pool length time, pool length count, stroke count, and stroke rate. Four video cameras were positioned on the pool edges to capture ground truth video footage, which was then manually labeled by three trained individuals. Mean (SD) differences between FORM Goggles and ground truth were calculated for the selected metrics for both sessions. The absolute mean difference and mean absolute percentage error were used to assess the differences of the FORM Goggles relative to ground truth. The test–retest reliability of the goggles was assessed using both relative and absolute reliability metrics.

**Results:**

Compared with video analysis, the FORM Goggles identified the correct stroke type at a rate of 99.7% (*N* = 2,354 pool lengths, *p* < 0.001), pool length count accuracy of 99.8%, and mean differences (FORM Goggles–ground truth) for pool length time: −0.10 s (1.49); stroke count: −0.63 (1.82); and stroke rate: 0.19 strokes/min (3.23). The test–retest intra-class correlation coefficient (ICC) values between the two test days were 0.793 for pool length time, 0.797 for stroke count, and 0.883 for stroke rate. Overall, for pool length time, the residuals were within ±1.0s for 65.3% of the total pool lengths, for stroke count within ±1 stroke for 62.6% of the total pool lengths, and for stroke rate within ±2 strokes/min for 66.40% of the total pool lengths.

**Conclusion:**

The FORM Goggles were found valid and reliable for the tracking of pool length time, pool length count, stroke count, stroke rate, and stroke type during freestyle, backstroke, and breaststroke swimming in recreational swimmers and triathletes when compared with video analysis. This opens perspectives for receiving real-time information on performance metrics during swimming.

## 1. Introduction

In endurance sports such as running and cycling, activity metrics are conveniently delivered to users in real time through interfaces such as bike computers, smartwatches, and tablets. As a mainstream fitness activity with 27.6 million active participants in the USA alone, according to the Outdoor Participation Report (1), swimming is, by contrast, poorly served by current technology. While data from smart watches and traditional manual timing equipment such as pool clocks and stopwatches are broadly available, this information is not accessible in real time because these interfaces are outside the line of sight of the swimmer while swimming. As such, swimmers commonly access performance metrics such as lap splits, distance, and pacing information between work bouts while they rest. It has previously been shown that real-time feedback assists pace control in swimming (2–5). Real-time feedback is also more effective compared with delayed feedback in running when it comes to pace control (6), running economy (7), and running technique (8–12). As such, it is plausible that receiving real-time information regarding relevant performance metrics during swimming could benefit performance. Along that line, using augmented reality in sports enables real-time performance analysis to better understand the development of motor skills in athletes (13).

Recently, a new category of tracking devices for swimming was introduced with the FORM Smart Swim Goggles (FORM Goggles, Vancouver, BC, Canada). The FORM Goggles have a built-in inertial measurement unit (IMU) and a see-through display and are capable of tracking and displaying distance, time splits, stroke, and pace metrics in real time using machine learning and augmented reality. These features set this device apart from other tracking solutions (14), because activity metrics are instantly available to the user through real-time visual feedback.

A systematic review of commercially available sensor technology for swimming published in 2015 (15) concluded that no published research material was available that investigated the accuracy, reliability, and validity of sensor-based methods for swimming performance analysis. To the best of our knowledge, only five studies published since 2015 have investigated the accuracy of both commercially available wrist-based and head-based tracking solutions (16–20). Finis Swimsense and Garmin Swim were evaluated against video analysis. Both devices accurately detected stroke type and distance, while lap time, stroke count, and stroke rate were not accurately captured. The latter metrics were, however, deemed acceptable for the needs of recreational swimmers (16).

The Apple Watch S2 and the Garmin Fenix 3 were evaluated for lap count and stroke count accuracy, and it was concluded that the Apple device was accurate to within 10% and the Garmin device was accurate to within 20% compared with manual count (17). The evaluation of a head-mounted sensor-based swim tracking device (TritonWear v1.2.3, 50 Hz, Toronto, ON, Canada) concluded that, compared with video analysis, it did not accurately measure distance, stroke count, velocity, or stroke type (20). By contrast, the same device did in fact accurately detect lap time, stroke count (19), and stroke rate (18). However, there were limitations in those studies related to the number of participants, swimming style, and swimming length.

Overall, the studies of commercially available wearable technology in swimming show that both wrist-based and head-based tracking devices provide a reasonable level of accuracy for recreational swimmers when it comes to pool length time, pool length count, stroke rate, stroke count, and stroke type. However, to our knowledge, there have not been any studies looking into the validity and reliability of wearable technology with visual real-time feedback modalities, nor have there been studies looking into tracking devices integrated into swimming goggles. Moreover, the development of devices providing real-time visual feedback in swimming also calls for studies that could show the benefits of such an approach for the athletes.

The purpose of the present study was to assess the validity and reliability of the FORM Goggles compared with video analysis for stroke type, pool length count, pool length time, stroke rate, and stroke count in recreational swimmers and triathletes. As shown by Mooney et al. (21), video analysis is a widely adopted method used to gather data for performance analysis in swimming.

## 2. Methods

### 2.1. Participants

A total of 36 participants were recruited [24 males, mean (SD): 35.5 (13.5) years; 177.7 (3.6) cm; 75.7 (7.4) kg, 12 females, 37.4 (16.1) years; 167.6 (3.7) cm; 65.6 (9.1) kg]. The inclusion criteria encompassed recreational swimmers and triathletes 18 years and above, who participated in pool swimming at least twice per month, swimming at least 1,000 m per pool session. The study participants were required to be proficient in backstroke (BK), breaststroke (BR), and freestyle (FR) by being able to swim continuously without stopping for 150 m for each stroke type. Given the technical challenge of swimming butterfly effectively, the stroke was removed as an inclusion criterion for the recruited recreational participants. The exclusion criteria encompassed currently active competitive swimmers to avoid biasing the results in favor of the highly regimented swimmers who are not the target market for the product, the participants with musculoskeletal injuries, and the participants residing outside the province of British Columbia (BC), Canada. The self-reported swim experience of the participants per stroke type was as follows: backstroke (27.8% beginner, 52.8% intermediate, 19.4% advanced), breaststroke (19.4% beginner, 58.3% intermediate, 22.2% advanced), and freestyle (2.8% beginner, 63.9% intermediate, 33.3% advanced).

All the participants were informed about the purpose of the study, and they gave a written informed consent to participate. The ethics approval was granted by Simon Fraser University (SFU) in BC, Canada (study number: 30000614). The participants were able to withdraw from the study at any point with no negative consequences, and an incentive of $100 CAD was given to the study participants who completed both swim session protocols.

### 2.2. Procedures

The study was carried out in a 25-m indoor swimming pool using two lanes with three participants in each lane for a total of six participants per session. Each session was split into two equal parts of 450 m each. Each set of 450 m was split into three intervals of four pool lengths of each stroke type (FR, BK, BR) with a 30-s rest in between and, additionally, by performing a set two times consisting of one pool length of each stroke type with 15-s rest in between (Table 1). In the first part of the session (SS1), the participants were instructed to swim at moderate effort corresponding to a rate of perceived exertion (RPE) of 3 out of 10 on the Borg CR10 scale (22, 23). In the second part of the session (SS2), the same participants were asked to swim the same workout, but at higher effort, corresponding to 7 out of 10 on the Borg CR10 scale. The exact same workout was repeated 7 days later in session two. The participants were pre-grouped based on age, gender, swimming background, and self-reported average pace per 100 m (if known). This grouping was done to avoid the incidence of participants passing each other during the prescribed swim sets. No incidence of hindrance of the regular swimming pace was observed in the swimmers in the same lane beyond the collisions and “stopping and pausing” exclusions listed in Table 2.

**Table 1:** Workout instructions per subject for session one and two with 1 week interval.

Swim session	Swim speed 1 (SS1)	Swim speed 2 (SS2)
Day 1 and 2	1 × 100 m FR, rest 30 s	1 × 100 m FR, rest 30 s
	1 × 100 m BK, rest 30 s	1 × 100 m BK, rest 30 s
	1 × 100 m BR, rest 30 s	1 × 100 m BR, rest 30 s
	2 × (25 m FR, 25 m BK, 25 m BR), rest 15 s	2 × (25 m FR, 25 m BK, 25 m BR), rest 15 s
Total distance per day = 900 m	450 m (+60 s rest)	450 m
Total pool lengths per day = 36	18	18

**Table 2:** Description of excluded pool lengths by type, criteria, and number.

Type	Criteria	Excluded pool lengths
Empty/missing data field	The participant paused the goggles too quickly at the end of the swim before the last pool length had been detected	16
Collisions	A collision occurs between the participants and disrupts normal swimming. If the collision happened within 4 windows (6 s) of the following pool length, then the following pool length was also removed	45
Poor technique	A pool length in which a participant has very poor stroke technique as defined by FINA Swimming Rules (24) and the USA officiating rules (25).	45
Stopping and pausing	The participant stops or pauses within a pool length or looks up/around, prior to end of pool length	31
Start/finish errors	The participant does not push off from the wall to start a pool length or does not touch the wall to end a pool length	36
Erratic behavior	The participant displays erratic behavior during rest intervals (i.e., active rest)	4
Stroke type switching	The participant switches stroke type within a pool length (except during backstroke flip turn)	3
Rest interval correction	Any pool length where rest interval was removed	46
Equipment failure	Goggles snapping off the participant's head, goggles combining two pool lengths into one	12
	Total pool lengths excluded:	238

To ensure sufficient time to offload the data from the goggles and the cameras, the entire data collection was carried out over 3 days with 12 participants per day and then repeated the following week for day 2 data collection at the same time of the day to avoid potential diurnal effects. This protocol resulted in 72 sessions of 900 m each for a total of 64,800 m or 2,592 pool lengths.

Before the data collection commenced, the participants were given a brief demonstration of the FORM Goggles with instructions on how to start and stop the swim session using the front button (Figure 2A) on the side of the device. The goggles were fitted using a correctly sized nose bridge, and the head strap tension was adjusted to avoid leaking during the trial. Ten minutes were allocated for familiarization and warmup in the pool before the formal test session began. For the warmup, the participants were instructed to swim 4 m  × 50 m intervals (FR, BK, BR, Choice) with a 10-s rest after each interval at a freely chosen pace.

Workout instructions were distributed to the participants by email prior to the swim sessions and written on a clearly visible whiteboard at the end of each lane. Once in the pool, the second author gave step-by-step workout instructions to each participant, and a printed version was placed inside a plastic folder on the pool deck at the end of each lane for easy reference during rest. Furthermore, the instructor explained the importance of following proper protocol for starts, turns (either flip or touch), and finishes for each stroke type and to continue swimming without stopping between turns even if being overtaken by a faster participant.

### 2.3. Equipment and setup

**Figure 1:**
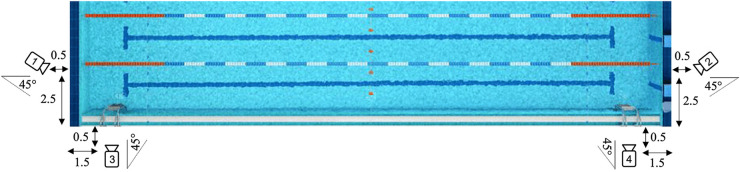
Placements of the video camera around the 25-m indoor pool.

Four waterproof 1080p video cameras (Hero 7, GoPro, San Mateo, CA, USA) were positioned on the pool edges, as shown in Figure 1. This camera setup was designed to capture ground truth video footage from six swimmers at a time, split across two lanes with three swimmers in each lane. Cameras 1 and 2 were positioned at a height of 1.0 m and placed 0.5 m from the end of the pool and 2.5 m from the side of the pool at an angle of 45° from horizontal level. These cameras captured footage of swim strokes in between turns for stroke type, stroke rate, and stroke count detection. Cameras 3 and 4 were designed to capture the swimmer starts, turns, and finishes for pool length time and pool length count detection. These cameras were placed at a height of 1.0 m and placed 0.5 m from the side of the pool and 1.5 m from the end of the pool at an angle of 45° from the horizontal level. The finest achievable measurement resolution for the cameras was 1 pixel per mm, and the theoretical limit for the time resolution was one frame every 33 ms (sampling frequency: 30 Hz). The camera angles covered both lanes and were synchronized at the start of each session and again multiple times during the post-session video analysis to account for potential camera drift, missing footage, or any other disturbances. Each swimmer was wearing a pair of FORM Smart Swim Goggles (FORM Goggles, Vancouver, BC, Canada) throughout the experiment as well as a unique color-coded swim cap for ease of identification during labeling.

**Figure 2:**
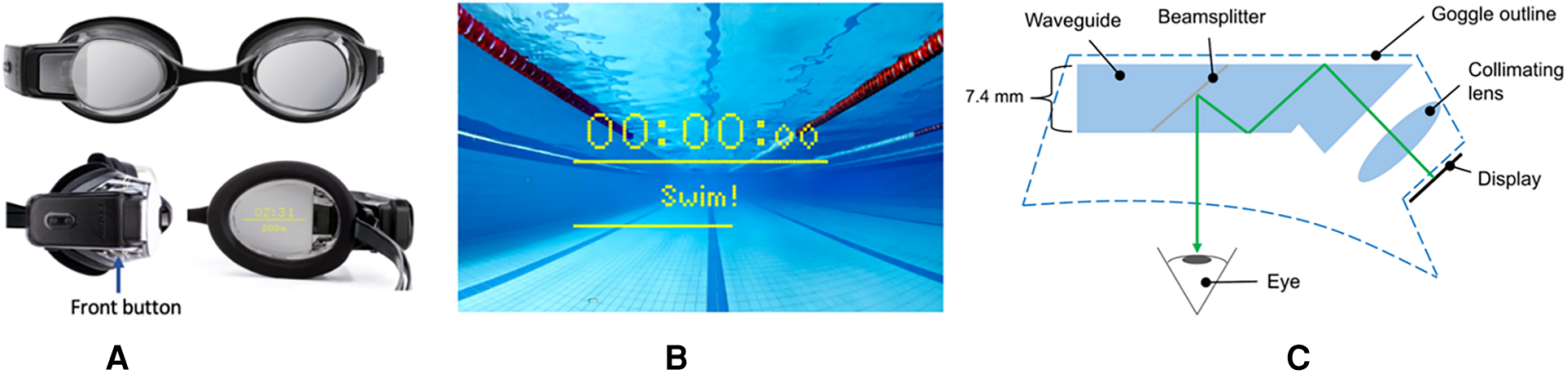
FORM Goggles (**A**), in-pool point-of-view (**B**), waveguide (**C**).

The FORM Goggles (Figure 2A) have an integrated microcomputer and display system. The microcomputer consists of a main processing chip, a data storage chip, a Bluetooth Low Energy chip, a battery, and a 9-axis Micro-Electromechanical System sensor. The 9-axis sensor combines a 3-axis accelerometer, a 3-axis gyroscope, and a 3-axis magnetometer into one IMU. This IMU detected the swim metrics using pre-trained machine-learning algorithms loaded onto the goggles. The resulting data from the IMU were fed to the display by the microcomputer in real time, allowing the swimmer to see the information while swimming.

The display system consists of an organic light emitting diode (OLED) micro-display and a see-through waveguide built from custom freeform optics integrated into one of the goggle lenses, as shown in Figure 2C. The display system can be set to show an augmented image split into two segments by a horizontal line (Figure 2B). The augmented image did not require the swimmer to refocus to read the information. The beam splitter inside the waveguide was designed to let in 50% of the ambient light and 50% of the light from the OLED display creating an augmented, mixed-reality viewing experience in which the swim metrics were visible without glancing down or to the side but appeared as if floating in front of the swimmer.

The FORM Goggles detect the following five swim metrics: stroke type, pool length time, pool length count, stroke count, and stroke rate (15). These metrics are detected by the onboard microcomputer and displayed in real time via the see-through display inside the goggles (Figure 2B).

Each pair of FORM Goggles used in the experiment was pre-configured to show the same metrics to each swimmer for each of the three swimming states (Swim, Turn, Rest). For the Swim state, “timer” and “pace per 100 m” were set as the top and bottom metrics and only visible in-between turns. For the Turn state, pool “length count” and pool “length time” were set and visible for 4 s after each turn. Finally, for the Rest state, “rest timer” and “pace per 100 m” were configured and only visible during rest.

The time-stamped swim data for each swimmer were saved on the FORM Goggles. The data were then uploaded into an anonymous form after each swim session to a secure and GDPR-compliant server via the FORM mobile phone app using a pin-protected Bluetooth Low Energy connection. Once in the cloud, the swim data were formatted and prepared for ground truth comparison using custom scripts written in Python.

### 2.4. Ground truth

The resulting video recordings were uploaded to a secure and private server. Here, the recordings were first analyzed for duplicate frames using the open-source software FFmpeg (FFmpeg developers, version 4.4.1, www.ffmpeg.org) and then synchronized and stitched together using Adobe Premier Pro (version 22.1.2, Adobe Inc., San Jose, CA, USA). The manual labeling was done using the open-source media player MPV (MPV Developers, version 0.34.0, http://www.mpv.io/) and conducted by three trained individuals following a script approved by the Director of Swim Coaching at Swim BC, the governing body for swimming in BC, and following generally established manual protocols (26–28). The labeled ground truth data and formats are shown in Table 3 and described in the sections below.

**Table 3:** Ground truth data formats for stroke type, pool length time, pool length count, stroke rate, and stroke count.

Metric	Format	Definition	Data type
Stroke type	FR, BR, or BK	Abbreviated stroke type for that pool length. FR: Freestyle, BK: Backstroke, BR: Breaststroke (butterfly and drills were excluded)	Categorical
Pool length time	Seconds	Time elapsed from push-off from one end of the pool until touch or turn at the other end of the pool	Float^a^
Pool length count	1,2,3…n	Number of pool lengths within the swim session	Integer
Stroke count	1,2,3…n	Number of strokes to complete one pool length	Integer
Stroke rate	Strokes/min	Number of strokes per minute	Float

^a^ Float, floating-point positive number.

#### 2.4.1. Starts, turns, and finishes

**Figure 3:**
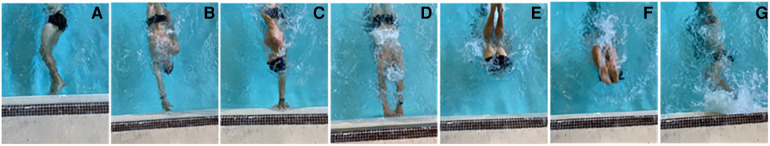
Starts (**A**), BK finish (**B**), FR finish (**C**), BR finish (**D**), flip turn (**E–G**), end-of-pool-length (**G**).

The ground truth pool length time (s) and pool length count metrics were labeled based on starts, turns, and finishes for each stroke type (Figure 3). The start of a pool length for all stroke types was measured from the feet leaving the wall, as shown in image A. The finish and touch-turn end-of-pool length for backstroke is shown in image B, for freestyle in image C, and for breaststroke in image D.

The flip turn sequences for backstroke and freestyle are shown in images E–G as seen from one of the two camera angles used (cameras 1 and 2). The end of a pool length for freestyle and backstroke flip turns was labeled as shown in image G (here, cameras 3 and 4 provide clear side views when the view is obstructed). This point in time was different from the start of a pool length in that the time split was labeled when the feet touched the wall vs. the feet leaving the wall, as shown in Figure 3A. This method reflects how the official Fédération Internationale de Natation-approved touchpad timing systems work for each stroke type during competitions (24).

#### 2.4.2. Stroke count, stroke rate, and stroke type

The stroke count for freestyle and backstroke was labeled based on individual strokes rather than stroke cycles (2 strokes = 1 cycle). Each time either arm made a propulsive pull, one stroke was counted. For breaststroke, the stroke count was labeled each time the head and arms came out of the water. In this instance, 1 stroke = 1 cycle since both arms moved together.

The stroke rate (26) for freestyle, backstroke, and breaststroke was measured using all the strokes counted in a pool length with the following formula:

Strokerate=(strokesinapoollength/strokingtime)∗60seconds

The stroking time for freestyle, backstroke, and breaststroke was measured from the start of the first pull to the end of pool length time. The first pull was defined as when the hands separated after streamline.

The manual recognition of stroke type by labelers was straightforward. The video footage clearly showed the swimmers performing either freestyle, backstroke, or breaststroke.

#### 2.4.3. Pool length exclusions

The labeled ground truth data were reviewed by the second author and the data team at FORM, and pool lengths were excluded when any of the eight criteria in Table 2 were met. The prescribed workout distance across sessions was 2,592 pool lengths with 238 exclusions resulting in a grand total of 2,354 pool lengths swum. The excluded pool lengths therefore represented 9.2% of the total number of pool lengths completed by the 36 participants across swim sessions.

### 2.5. Comparison of FORM Goggles data with ground truth data

A custom script was written in Python (version 3.10.0) to extract the data from the sensor files uploaded from the FORM Goggles and compared with the corresponding ground truth data. The sensor files and the ground truth files were matched using the timestamps for each file, and the resulting differences between the five variables measured in the study represented the residuals used for the statistical analysis.

## 3. Statistical analysis

The mean (SD) differences between the FORM Goggles and ground truth were calculated for pool length time, pool length count, stroke count, and stroke rate for both sessions. The absolute mean difference and mean absolute percentage error (MAPE) were used to assess the differences of the FORM Goggles relative to ground truth, and the standardized effect (i.e., mean difference by pool length divided by the pooled SD) determined the size of this difference (0.2–0.5 = “small,” 0.5–0.8 = “medium,” and >0.8 = “large”) (29). The agreement between Form Goggles values and ground truth values was quantified using the Bland–Altman limits of agreement (30). This statistical method was applied under the assumption of normality for the distribution of residual values for pool length time, stroke count, and stroke rate for each stroke type. A linear regression analysis was performed to measure any proportional bias for pool length time, stroke count, and stroke rate (31). The agreement between Form Goggles and ground truth for pool length count was determined by subtracting the total number of miscounted pool lengths from the total pool lengths and dividing by total pool lengths. A Fisher exact test was applied to determine the percentage count frequencies across all four stroke types (including butterfly) for both the FORM Goggles and ground truth. The reliabilities of the video raters (intra-rater, inter-rater) and the goggles (test–retest) were assessed using both relative and absolute reliability metrics (32, 33). The intra-class correlation coefficient (ICC) was used as the metric of relative reliability, with the specific versions of the ICC specified as follows (34): first, the intra-rater reliability of the three ground truth labelers to label the same video footage consistently using a two-way, mixed-effects, absolute agreement, single measure model ICC (3,1); second, the inter-rater reliability of the three ground truth labelers to label the same video footage consistently between them using a two-way, random effects, absolute agreement, single measure model ICC (2,1). The specific data used for these ICCs were generated from a random selection of four pool lengths of each stroke type sampled from the data of ten randomly sampled participants.

Third, the test–retest reliability of the Form Goggles to produce similar results across the two test sessions for pool length time, stroke count, and stroke rate was assessed by computing the ICCs on the residuals for sessions one and two using a one-way, mixed-effects, absolute agreement, multiple measurements ICC (1, k) (35). Since the data were unbalanced due to the exclusion criteria, and the trial variances were zero, a linear mixed-effects model was used to estimate the variance components for the ICC formulae (33). The ICCs were then interpreted according to Landis and Koch (36) where values between 0.00–0.40 indicate unacceptable agreement, 0.41–0.60 moderate agreement, 0.61–0.80 substantial agreement, and 0.81–1.00 almost perfect agreement.

The standard error of the measurement (SEM) was calculated to assess the test–retest reliability of the goggles in absolute terms (33). The SEM was then used to estimate the minimal detectable change (MDC) with a confidence interval of 95% (33). All statistical calculations were performed using R (version 4.2.0, R Core Team, Vienna, Austria).

## 4. Results

The ICC coefficients for the intra-rater and inter-rater reliabilities in Table 4 were all above 0.81, ranging from 0.96 to 1.00 with the lowest scores for stroke rate and the highest scores for pool length time and pool length count. These results indicate “almost perfect agreement” between repeated measures for all swimming metrics.

**Table 4:** Intra-rater and inter-rater intra-class correlation (ICC) coefficients per metric and stroke type (33, 34).

Variable	ICC (intra-rater)	ICC (inter-rater)
Model	Two-way, mixed-effects, absolute agreement, single measure (3,1)	Two-way, random effects, absolute agreement, single measure (2,1)
Pool length time (s)		
All	0.997	0.999
Backstroke	0.992	1.030
Breaststroke	1.000	0.999
Freestyle	1.000	0.999
Stroke count (strokes/pool length)		
All	0.997	0.997
Backstroke	0.994	0.996
Breaststroke	0.999	0.994
Freestyle	0.997	0.995
Stroke rate (strokes/min)		
All	0.994	0.995
Backstroke	0.959	0.974
Breaststroke	0.994	0.987
Freestyle	0.987	0.982
Pool length count		
All	1.000	1.000
Backstroke	1.000	1.000
Breaststroke	1.000	1.000
Freestyle	1.000	1.000

Table 5 shows the classification accuracy of the stroke type of the FORM Goggles compared with ground truth for each of the four strokes. Overall, the FORM Goggles identified the correct stroke type at a rate of 99.7% (*N* = 2,354, *p* < 0.001). Freestyle (*N* = 832) and Backstroke (*N* = 734) were correctly classified across all pool lengths swum (*p* < 0.001), while breaststroke was misclassified as butterfly for seven pool lengths corresponding to a butterfly false positive rate of 0.9% (*N* = 788, *p* = 1.00).

**Table 5:** Agreement (classification accuracy) between FORM Goggles and ground truth for stroke type across all pool lengths (*N* = 2,354).

	Butterfly	Backstroke	Breaststroke	Freestyle
Butterfly (ground truth)	0.0% (0)	0.0% (0)	0.0% (0)	0.0% (0)
Butterfly (FORM Goggles)	0.0% (0)	0.0% (0)	0.0% (0)	0.0% (0)
Backstroke (ground truth)	0.0% (0)	100% (734)	0.0% (0)	0.0% (0)
Backstroke (FORM Goggles)	0.0% (0)	100% (734)	0.0% (0)	0.0% (0)
Breaststroke (ground truth)	0.0% (0)	0.0% (0)	100% (788)	0.0% (0)
Breaststroke (FORM Goggles)	0.9% (7)	0.0% (0)	100% (781)	0.0% (0)
Freestyle (ground truth)	0.0% (0)	0.0% (0)	0.0% (0)	100% (832)
Freestyle (FORM Goggles)	0.0% (0)	0.0% (0)	0.0% (0)	100% (832)

Figures in parenthesis indicate number of pool lengths registered per stroke type and method of measurement.

The mean differences per pool length between the FORM Goggles and ground truth for pool length time, stroke count, and stroke rate are shown in Table 6 for each stroke type and overall. Five pool lengths out of the 238 pool length exclusions in Table 2 were incorrectly counted by the goggles and not caused by user error, corresponding to a pool length count accuracy of 2,354/(2,354 + 5) = 99.79%. The mean differences (FORM Goggles–ground truth) for the other three metrics measured were the following: pool length time: −0.10 s (1.49), stroke count: −0.63 (1.82), and stroke rate: 0.19 strokes/min (3.23). The standardized effects were small (i.e., <0.5) for all measurements across metrics and stroke types. The test–retest ICC values across LT, SC, and SR between the two test days were all above 0.60 ranging from “substantial agreement” to “almost perfect agreement” except for pool length time (breaststroke) and pool length time (backstroke), which both showed moderate agreement. The SEM for pool length times was 1.1 s or less, which corresponded to MDC of 1.8 s to 3.0 s.

**Table 6:** Validity and reliability of the FORM Goggles compared with ground truth [mean (SD)].

	Mean difference	Abs. mean difference	MAPE (%)	Std. effect	Test-retest		
					ICC	SEM	MDC
Pool length time (s)							
All	−0.10 (1.49)	1.01	3.40	0.01	0.793	0.677	1.877
Backstroke	0.03 (1.62)	1.09	3.37	0.00	0.603	1.020	2.827
Breaststroke	−0.15 (1.68)	1.22	3.84	0.02	0.562	1.114	3.087
Freestyle	−0.17 (1.12)	0.75	3.02	0.03	0.610	0.699	1.937
Stroke count (strokes/pool length)							
All	−0.63 (1.82)	1.36	6.40	0.09	0.797	0.821	2.277
Backstroke	−0.49 (2.15)	1.69	7.24	0.06	0.842	0.856	2.373
Breaststroke	0.04 (1.11)	0.63	4.02	0.01	0.789	0.509	1.412
Freestyle	−1.40 (1.77)	1.78	7.90	0.27	0.800	0.794	2.199
Stroke rate (strokes/min)							
All	0.19 (3.23)	1.83	3.90	0.01	0.883	0.910	2.522
Backstroke	0.30 (3.97)	2.20	4.40	0.04	0.854	1.119	3.102
Breaststroke	0.75 (1.68)	1.22	4.00	0.15	0.793	0.765	2.120
Freestyle	−0.44 (3.50)	2.07	3.37	0.05	0.857	1.136	3.149

Mean (SD) differences (FORM Goggles–Ground truth), absolute mean difference, mean absolute percentage error (MAPE), standardized effect (std. effect), test–retest ICC, standard error of the mean (SEM), and minimal detectable change (MDC) for the swimming metrics across all valid pool lengths (*N* = 2,354) (33–35).

**Figure 4:**
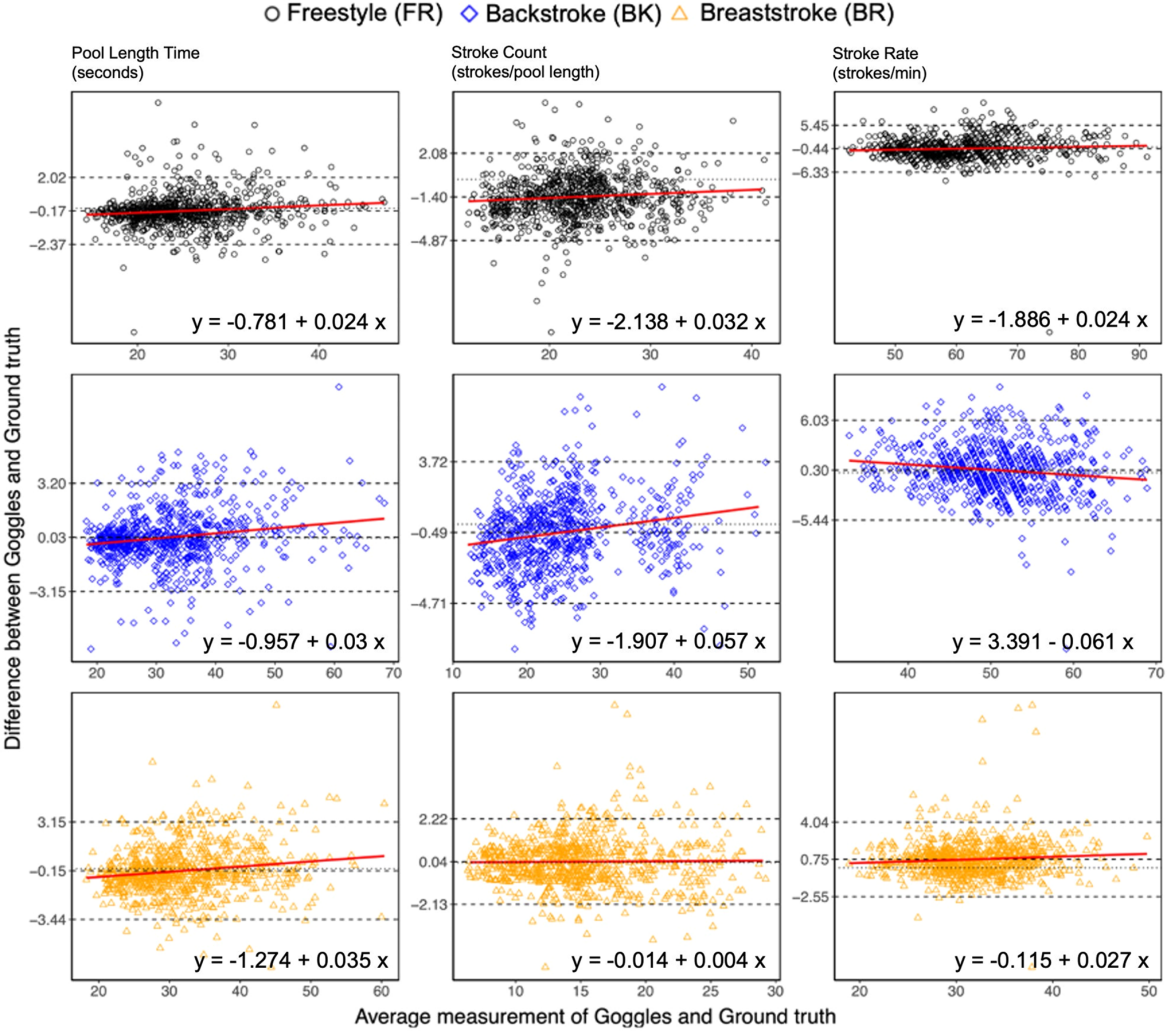
FORM Goggles—ground truth by pool length for pool length time, stroke count, and stoke rate. Dotted lines: zero difference reference, dashed lines: bias and upper/lower limits of agreement (95% confidence interval), solid lines (with equations): linear regression (37, 38).

The Bland–Altman plots for pool length time, stroke count, and stroke rate for all pool lengths (*N* = 2,354) are shown in Figure 4. The linear regression analysis (31) showed that residual scores had a small positive proportional bias with the following regression coefficient ranges (pool length time: 0.0241–0.0351, stroke count: 0.0325–0.0571, and stroke rate: 0.0238–0.0271) across metrics and stroke types with three exceptions. For stroke rate (backstroke), there was a small negative proportional bias (−0.0611), and for stroke count (Breaststroke) and stroke rate (Freestyle), the bias was constant. On average, the FORM Goggles showed shorter pool length times than ground truth for freestyle (−0.17 s, LOA: −2.37 s to +2.02 s) and breaststroke (−0.15 s, LOA: −3.44 s to +3.15 s) while for backstroke, the pool length times were slightly longer than ground truth (0.03 s, LOA: −3.15 s to +3.21 s). For stroke count, the FORM Goggles under-counted compared with ground truth for freestyle (−1.41 strokes, LOA: −4.99 to +2.16) and backstroke (−0.53 strokes, LOA: −5.07 to +4.02) while slightly over-counting for breaststroke (0.04 strokes, LOA: −2.13 to +2.22). For stroke rate, the FORM Goggles were under-counting compared with ground truth for freestyle (−0.5 strokes/min, LOA: −7.36 to +6.35) while over-counting for backstroke (0.2 strokes/min, LOA: −7.59 to +7.98) and breaststroke (0.75 strokes/min, LOA: −2.55 to +4.04).

Overall, for pool length time, the residuals were within ±1.0 s for 65.3% of the total pool lengths, for stroke count within ±1 stroke for 62.6% of the total pool lengths, and for stroke rate within ±2 strokes/min for 66.40% of the total pool lengths. Freestyle was the most accurate stroke type for pool length time, with 76.20% of pool lengths within ±1.0 s, whereas breaststroke was the most accurate stroke type for stroke count (87.40% within ±1 stroke) and stroke rate (84.40% within ±2 strokes/min).

## 5. Discussion

This study evaluated the validity and reliability of FORM Smart Swim Goggles compared with video analysis for the variables pool length time, pool length count, stroke count, stroke rate, and stroke type over a total of 2,354 pool lengths. The high intra-rater and inter-rater reliability scores showed high consistency within and between video labelers in the study. The results also showed significant agreement between FORM Goggles and ground truth for all metrics across backstroke, breaststroke, and freestyle, respectively. Reliabilities across the two sessions were “substantial” to “almost perfect” agreement for all variables except in breaststroke and backstroke for pool length time where the ICC showed “moderate agreement” (ICC: 0.562–0.603). The reported MDC will enable improvements in the assessment of performance for any subsequent randomized controlled trials.

The FORM Goggles performed as well or better than existing swimming wearable technologies (16–20). The ability of the FORM Goggles to correctly detect stroke type contrasted with results from previous studies using head-mounted sensors (20, 39), in which accurate detection of stroke type was significantly impaired. Wrist-mounted sensors have been shown to provide better results for stroke type (16), especially for backstroke and freestyle (39). The results for pool length count accuracy were comparable with previous studies based on wrist-based tracking solutions from Garmin, Apple, and Finis (16, 17), whereas pool length time, stroke count, and stroke rate accuracies were better than the leading wrist-based solutions from Garmin and Finis (16). For these studies, the best-performing device produced MAPE across stroke types of 9.03% (LT), 14% (SC), and 13.3% (SR) compared with 3.41% (LT), 6.48% (SC), and 3.98% (SR) for the FORM Goggles. A recent study in 2021 (19) on a head-mounted IMU from Triton Wear revealed results for pool length time, stroke count, and stroke rate that were similar to the FORM Goggles. However, Pla et al. (19) only enrolled a homogenous group of six swimmers consisting of elite open water swimmers, which limited the generalizability of their findings. In general, most studies performed on IMUs in swimming have been conducted on competitive swimmers to ensure reliable and consistent performances of the participants and therefore represent the best case in terms of minimizing participant errors (14). The use of non-competitive swimmers in our study produced biases across metrics and stroke types that were low and residuals that were uniform (Figure 4). Furthermore, our results generalize to non-competitive swimmers and suggest that they could benefit from using FORM Goggles. This suggestion is substantiated by the fact that technology can improve visual information uptake (40).

The accuracy of the FORM Goggles to produce valid results is a function of the quality of the machine learning algorithms used to predict outcomes based on head movements and the size and diversity of the populations used in data collection when training these algorithms (41). The training data for the FORM Goggles encompassed swimmers of varying abilities, ages, and genders with the aim of achieving a high level of accuracy across all populations. The complexity of the movements involved in the prediction of a given variable also plays a role. For example, detecting a turn is simpler than detecting strokes, which was evidenced by the very high pool length count accuracy with only 0.2% error compared with stroke count accuracy, where MAPE ranged from 4.0% to 7.9%. Conversely, counting strokes for breaststroke is simpler than counting strokes for freestyle and backstroke since the arms for these strokes move separately through the water resulting in two strokes per stroke cycle. Similarly, the participants swimming freestyle and backstroke often perform half strokes when coming into the wall before the turn and when breaking through the water after the turn. This results in cases where the FORM Goggles are not counting half strokes as full strokes, but where ground truth labelers are more conservative and do count these strokes. As a result, breaststroke had the lowest MAPE (4.02%) of the three stroke types for stroke count and had the smallest bias (0.04) compared with freestyle (−1.40) and backstroke (−0.49), respectively.

The reliability of the FORM Goggles to produce similar results between the two test sessions was good. This was evidenced by the high ICC scores despite low between-subject variability as shown in the Bland–Altman plots in Figure 4 (33, 42), which generally causes lower ICC scores (33). The FORM Goggles are therefore reliable when it comes to producing consistent predictions for metrics across swim sessions for recreational swimmers and triathletes. The test–retest reliability results could not be compared against other studies of commercially available tracking devices for swimming since reliability was not measured in those studies (16, 17, 19, 20). Further, the computed SEM and MDC values will provide a basis for studying the effect of using the FORM Goggles to enhance swimming performances.

The swimmers who are using swim trackers without a real-time feedback modality are not aware of device errors unless they are at rest or have completed their swims. Even then, only the most glaring mistakes are caught, such as false pool length counts and timing errors that fall outside the inherent inaccuracy of sighting and manually calculating interval times from the pool clock. The same cannot be said about swim trackers with a real-time display because metrics are visible to the swimmer without stopping. Pool length count and stroke count are easy to verify, while stroke rate and pool length time require more concentration, since sighting the pool clock correctly while moving is not trivial. Close to two-thirds of all pool lengths had errors that fell within ±1 s for pool length time, ±1 strokes for stroke count, and ±2 strokes/min for stroke rate. For freestyle, which is the most common stroke type used in training (14, 16), more than 75% of pool lengths were within ±1 s from ground truth for pool length time. In swimming, similar to running, pacing and time splits are central to monitoring performance (3, 43, 44). Taken together, the FORM Goggles was found as a valid device for monitoring real-time performance for recreational swimmers and triathletes when it comes to pool length time, pool length count, stroke count, stroke rate, and stroke type.

This study features the following noteworthy limitations. First, we chose a 25-m pool to maximize the number of pool lengths per distance swum and because most public lap pools are either 25 m or 25 yd long. Thus, data were not collected in a 50-m pool even though this pool length is also widely used. While results for stroke type, stroke rate, and pool length count would have been comparable, MAPE for pool length time and stroke count would have been smaller since the denominators in calculating MAPE for these metrics would have been twice as large. However, the four comparable validity studies for commercially available swim trackers also only collected data in 25-m pools, so comparing results with these widely used swim trackers was sound (16, 17, 19, 20). The 238 erroneous pool lengths (Table 3) were in part caused by the environment (collisions, starting/stopping, etc.) and in part by the behavior of the participant, where three beginner swimmers generated 65% of these participant errors with the majority occurring in backstroke. The misclassification of seven butterfly pool lengths, however, was caused by one advanced swimmer, as a powerful breaststroke technique can closely resemble the butterfly, with both the head and body forcefully emerging out of the water. In the field, many beginners would likely experience similar errors at times although learning effects would reduce many of the error occurrences after a few swims since adjustments to behavior can be made quite easily when errors are visible in real time. This was also evident in the error split across the two swim sessions where approximately 60% of the errors were generated in session 1 and only 40% in session 2.

## 6. Conclusion

In conclusion, the FORM Goggles were valid and reliable for the tracking of pool length time, pool length count, stroke count, stroke rate, and stroke type during freestyle, backstroke, and breaststroke swimming in recreational swimmers and triathletes when compared with video analysis. The accuracy was better than the widely used commercial devices on the market targeting recreational swimmers and triathletes including a device marketed to competitive swim teams. The FORM Goggles offer important perspectives for swimmers who are willing to improve their swimming style and overall swimming performances.

## Data Availability

The original contributions presented in the study are included in the article/Supplementary Material; further inquiries can be directed to the corresponding author.
